# 

**Published:** 1917-06

**Authors:** 


**1000 Procrastinators ! ! ! Are You One ?**

*Doctor Somebody:*

There are about 1000 doctors on the subscription list of the TEXAS MEDICAL JOURNAL who owe me the little sum of $1.00 each, but a dollar multiplied by one thousand makes a big sum to a woman who has to depend on *your dollar* to enable her to get out a journal.

The chances are more than even that you are one of that 1000 who have been expecting to pay tomorrow or the next day or next week, but just haven't paid it yet. Supposin', now supposin', you sit right down and send along *my dollar,* for if you put it off again, you may forget it, and I really need the money.

I know you intend to pay it, just like you expect those who owe you to pay, but it is such a little amount that you have neglected it. Because it is small it makes it so much harder for me to collect, for if it was $100 you would be wanting to get it settled right away .

It costs money to send out bills, for stamps and stationery must be paid for, so don't wait for a bill or another if you have already had one.

Now, please send it along today in the way most convenient for you to send it, and you will cut the number of dilatory subscribers down just that much.

TEXAS MEDICAL JOURNAL.

P. S.—If you are not behind, the dollar will pay your subscription just that much in advance, and will be just as much appreciated. You may make it $2.00 or $3.00 if you wish, and get credit for whatever you send, thus keeping me from reminding you of it again soon.

